# Enhancing Self-Efficacy for Help-Seeking Among Transition-Aged Youth in Postsecondary Settings With Mental Health and/or Substance Use Concerns, Using Crowd-Sourced Online and Mobile Technologies: The Thought Spot Protocol

**DOI:** 10.2196/resprot.6446

**Published:** 2016-11-04

**Authors:** David Wiljer, Alexxa Abi-Jaoude, Andrew Johnson, Genevieve Ferguson, Marcos Sanches, Andrea Levinson, Janine Robb, Olivia Heffernan, Tyson Herzog, Gloria Chaim, Kristin Cleverley, Gunther Eysenbach, Joanna Henderson, Jeffrey S Hoch, Elisa Hollenberg, Huan Jiang, Wanrudee Isaranuwatchai, Marcus Law, Sarah Sharpe, Tim Tripp, Aristotle Voineskos

**Affiliations:** ^1^ University Health Network Toronto, ON Canada; ^2^ Centre for Mental Health and Addiction Toronto, ON Canada; ^3^ University of Toronto Toronto, ON Canada; ^4^ Centre for Global eHealth Innovation Toronto, ON Canada; ^5^ Centre for Excellence in Economic Analysis Research (CLEAR) St. Michael’s Hospital Toronto, ON Canada; ^6^ Department of Public Health Sciences University of California Davis, CA United States; ^7^ Cancer Care Ontario Toronto, ON Canada; ^8^ Michael Garron Hospital Toronto, ON Canada; ^9^ QoC Health Toronto, ON Canada

**Keywords:** mental health, substance use, help-seeking, participatory action research, eHealth, mobile applications, crowd-sourcing, transition-aged youth

## Abstract

**Background:**

Seventy percent of lifetime cases of mental illness emerge prior to age 24. While early detection and intervention can address approximately 70% of child and youth cases of mental health concerns, the majority of youth with mental health concerns do not receive the services they need.

**Objective:**

The objective of this paper is to describe the protocol for optimizing and evaluating Thought Spot, a Web- and mobile-based platform cocreated with end users that is designed to improve the ability of students to access mental health and substance use services.

**Methods:**

This project will be conducted in 2 distinct phases, which will aim to (1) optimize the existing Thought Spot electronic health/mobile health intervention through youth engagement, and (2) evaluate the impact of Thought Spot on self-efficacy for mental health help-seeking and health literacy among university and college students. Phase 1 will utilize participatory action research and participatory design research to cocreate and coproduce solutions with members of our target audience. Phase 2 will consist of a randomized controlled trial to test the hypothesis that the Thought Spot intervention will show improvements in intentions for, and self-efficacy in, help-seeking for mental health concerns.

**Results:**

We anticipate that enhancements will include (1) user analytics and feedback mechanisms, (2) peer mentorship and/or coaching functionality, (3) crowd-sourcing and data hygiene, and (4) integration of evidence-based consumer health and research information.

**Conclusions:**

This protocol outlines the important next steps in understanding the impact of the Thought Spot platform on the behavior of postsecondary, transition-aged youth students when they seek information and services related to mental health and substance use.

## Introduction

The onset of mental illness across the lifespan is highest among children and youth, with 70% of cases emerging before age 24 [1]. Despite the known benefits of early identification and treatment, evidence shows that at-risk members of this age group have great difficulty accessing and receiving the kinds of mental health services they need [2-6]. Untreated mental health concerns in emerging adulthood increase the risk of enduring mental illness, and are also associated with greater risk of dropping out of school, unemployment, youth justice involvement, bullying, traumatic release from care, and self-medication with alcohol and other drugs [7,8]. Most alarmingly, suicide is the second leading cause of death among Canadian youth, and is attributed to 20% of all deaths among young adults aged 15 to 24 [7]. Studies have reported that 10% of students have considered suicide at least once during their time at university or college [9-11].

The current social and medical system makes the act of help-seeking a relatively unlikely choice for many transition-aged youth (for the purposes of this study we define transition-aged youth as those aged 16-29 years) [3,4,12,13]. Youth in Ontario experience barriers in accessing appropriate services, information, and advice because the system is “fragmented, spread across several ministries, and offered in a variety of care settings” [14]. The barriers associated with help-seeking in young adults vary, and are highest when there is lower access to health services, less support from family and friends, and a lower sense of self-worth [15]. The stigma attached to mental illness, as well as embarrassment and fear of confidentiality breaches, are other major barriers associated with help-seeking among young adults [6,11,16-20].

Mental health literacy, or “knowledge and beliefs about mental disorders which aid their recognition, management, or prevention” [21], is generally poor among transition-aged youth, with many unable to identify the signs of a mental health problem or determine when professional help is needed [11,22,23]. When youth do seek mental health information, they do so through a *needs-driven approach*, typically searching for information only when there is a specific purpose [24]. Research shows that youth prefer informal avenues of information and support such as friends, family, or significant adults, rather than more formal sources such as psychologists, psychiatrists, or family doctors [6,17,20,25]. This informal help-seeking behavior can, however, result in risks; these sources are not always equipped with sufficient knowledge or skills to provide mental health support or information.

Enabling youth to seek and access mental health services is vital to the sustainability of a health care system [25]. With more than 90% of youth using the Internet [26-28], Web-based electronic health (eHealth) and mobile-based health (mHealth) interventions have been identified as promising tools for reaching this age group [11,27,29-32], potentially bridging the gap for hard-to-reach populations such as young males who have been reluctant to seek services [22,33,34]. The online environment provides transition-aged youth with the “opportunity to interact with, share, view, and access information through a number of convenient formats” [35,36]. Websites that are easy to navigate and visually engaging are preferred by many young people [24], while interactive elements, such as coaching and crowd-sourcing, are effective and well-received [25]. These less-formal or less-specialized online or mobile mental health interventions and resources have been shown to be effective avenues to engage transition-aged youth [25,30,31,37-41] and enhance “their capacity to correctly recognize, identify, and receive help for psychological disorders in a manner that is both accessible and nonthreatening” [15]. Such interventions can cross geographic barriers, reduce wait times, provide anonymity, and actively engage youth in facilitating mental well-being [13,41-43].

Youth-driven projects have been shown to increase participation in mental health care, better address youth concerns, and produce more relevant outcomes [44-47]. However, there are currently few solutions (cocreated with youth and health professionals that address existing needs and gaps) that are able to integrate evidence and experience. This project, driven and cocreated by transition-aged youth in postsecondary settings, will enhance and test an open-source eHealth and mHealth intervention (Thought Spot) that aims to involve their peers and enable them to identify and overcome barriers to obtaining help for mental health-related issues. Thought Spot is designed to improve attitudes and enhance self-efficacy for help-seeking for mental health support and services, and thereby increase the utilization of appropriate mental health and wellness services.

The objective of this paper is to describe the protocol for optimizing and assessing Thought Spot, a Web- and mobile-based platform cocreated with end users, that is designed to improve the ability of transition-aged youth in postsecondary settings to access mental health services.

## Methods

### Theoretical Basis and Protocol Implementation

Drawing on social-cognitive theory as a guiding conceptual construct, this study will investigate the impact of crowd-sourced, socially constructed, and evidence-informed online and mobile technologies on *self-efficacy* to access and utilize mental health and wellness services. Social-cognitive theory recognizes individuals as being self-organizing, proactive and self-reflective [48-50] and conceptualizes self-development, adaptation, and change as being embedded within a broad network of socio-structural influences and psychological factors [28,51-53]. This study will draw from a theory of help-seeking that proposes a 4-stage process: awareness, expression, availability and willingness [22]. The process begins with an awareness of symptoms and the appraisal of having a problem that may require intervention. This awareness must then be articulated or expressed by the help-seeker in plain language that can be understood by others, and sources of help must be available and accessible [22]. Finally, the help-seeker must be willing and able to access support and disclose their concerns [22].

### Thought Spot Developmental Process: Cocreation

The Thought Spot platform was originally developed through the Mental Health Innovation Fund, a program established by the Ontario Ministry of Training, Colleges and Universities. While the original collaboration included the University of Toronto’s Faculty of Medicine (the lead organization), the Centre for Addiction and Mental Health (CAMH), Ryerson University, Ontario College of Art and Design (OCAD) University, and ConnexOntario, the project was always envisioned as a student-led innovation project focused on improving postsecondary students’ access to, and navigation within, mental health and addiction services. To achieve this goal, the idea of a digital map of *all* addiction and mental health services, starting with the Greater Toronto Area (GTA; Ontario, Canada), was proposed. This map would be developed in partnership with students, educators, and health service providers. It was envisioned that the map would respond effectively to the challenges presented by an often-fragmented system of mental health services available to students (eg, on-campus services and community-based services are funded separately, making navigation between services challenging). Creating an online mapping platform was seen as a way to increase access and ease of navigation, and to address other barriers such as stigma and a lack of mental health literacy. It was proposed that the scope of the map would be informed by a *social determinants of health* perspective, and its dissemination would be through Internet and mobile devices. The aim of the strategy was to support direct help-seeking intentions, attitudes, and behaviors by increasing knowledge of appropriate services for postsecondary students, and minimizing the need for intermediaries (ie, friends, family, family physician).

Project leaders at CAMH identified an open-source software (Ushahidi) that could both display mapped information and allow for crowd-sourcing, a strategy aimed at supporting the sustainability of the Thought Spot platform and allowing for the participation of youth in its development. Second, the project team initially *seeded* the map with data supplied by collaborating organization Kids Help Phone and project partner ConnexOntario. From that starting point, the team used a participatory model of engagement to work with 65 university and college students to develop the platform. The students were recruited from institutions across the GTA to work on all aspects of the project. Students were organized into 2 teams, focusing respectively on production and development, to lead the development of the digital intervention, as well as several working groups that focused on specific aspects of the project, including design, promotion, and knowledge translation. The participatory approach defined specific roles for student leadership and student decision-making. A project steering committee balanced student and organizational input, enabling students to make a wide variety of key decisions on issues such as the name of the project, the logo, project management, and product design. This work included the organization of the original *seeded* mental health resource data with a taxonomy, which became the basis for categories through which users would enter the map and/or filter the data. The categories include health and wellness, sex and relationships, legal and financial, work and school, spirituality and well-being, and friends and family. Through this work the students developed Thought Spot as a platform that aimed to:

1. Mobilize other students to share their knowledge about services.

2. Discover wellness options in their area.

3. Build peer networks.

4. Read reviews and comments from peers about services.

5. Add their own *thought spots* or services.

In order to create a mobile version of Thought Spot, the project hosted Hackathought, an open digital hackathon event in Toronto in November 2014. This work led to the development of a native mobile app (iOS and Android), as well as a *responsive* design of the Thought Spot website, which allows for optimal viewing across a range of devices (eg, desktop computer monitors, tablets, and mobile devices). These platforms were all completed in March 2015.

### Thought Spot Functionalities

User-generated information has been entered in the Thought Spot platform for over 1000 mental health and wellness *spots* (eg, campus health centers, mental health and substance use services, peer support, crisis information, public parks, libraries, yoga studios). The platform has been further developed through a series of workshops in which teams of students verified the *spots* and customized them for peers seeking services, adding commentary and information that students would want to know in language that speaks directly to them.

**Figure 1:**
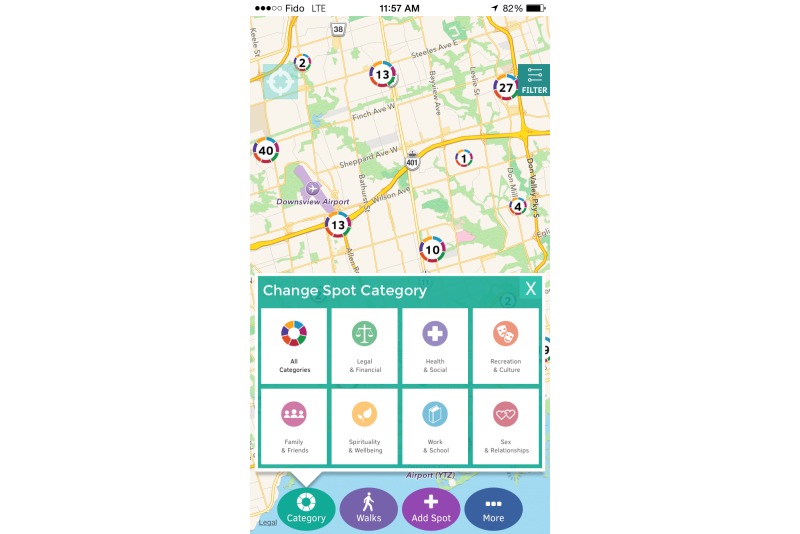
Users can use the Category section to locate specific types of spots in their surroundings.

**Figure 2:**
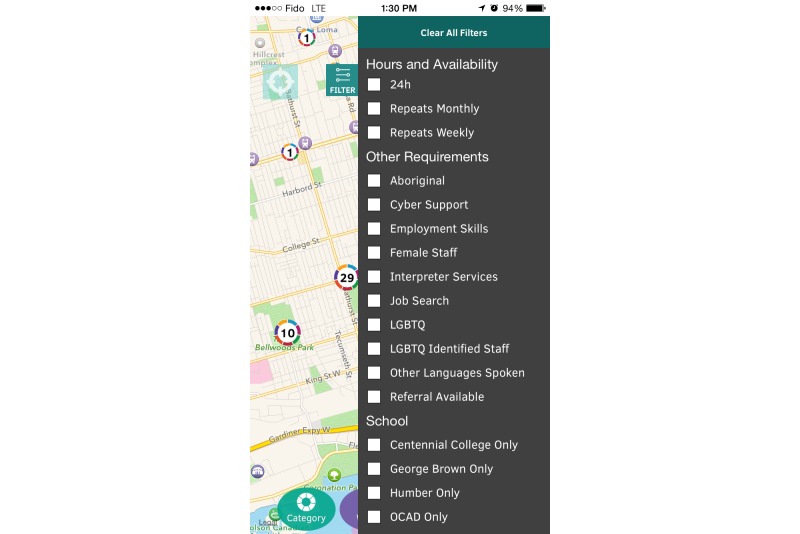
To further narrow down search results, users can apply filters to their searches. Only locations that fit within the filters will be displayed on the map.

**Figure 3:**
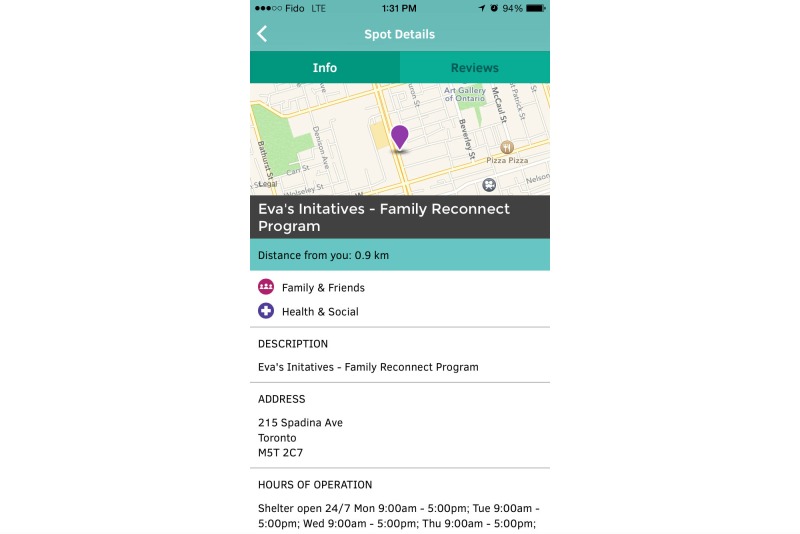
When users select a certain spot on the map, they will be provided with the spot’s details. Users can either get general information about the location, or see reviews left by other users.

The main functionality of the Thought Spot platform allows users to geo-locate themselves and search for *spots* through an interactive map (Figure 1). *Spots* can be searched according to cost, hours, catchment, and other parameters such as the availability of specific lesbian, gay, bisexual, transgender, transsexual, two-spirit, intersex, questioning, queer, and Aboriginal services (Figure 2). Once an appropriate spot has been located, users can access more specific information about the service (such as accessibility, languages offered, and wait list information), allowing them to decide whether the service is right for them (Figure 3).

**Figure 4:**
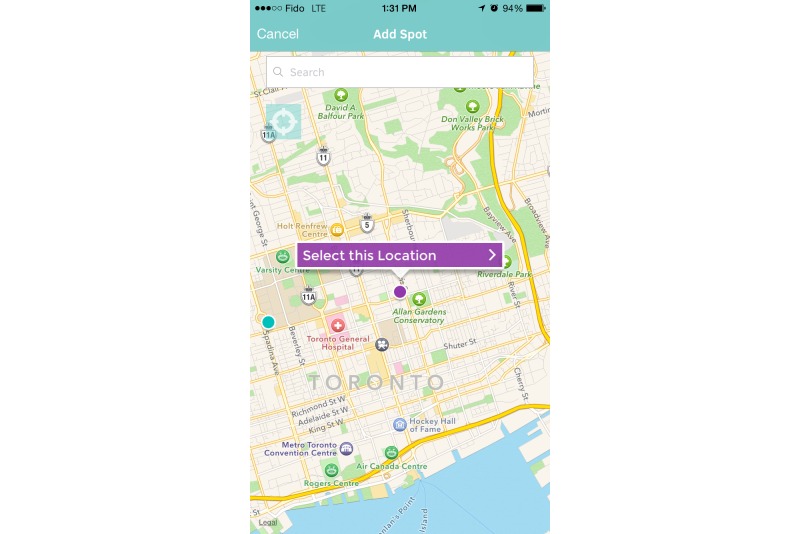
Users can contribute to the Thought Spot community by adding their own mental health and/or well-being spots. These spots are reviewed and assessed by members of the Thought Spot team before becoming available to the public.

**Figure 5:**
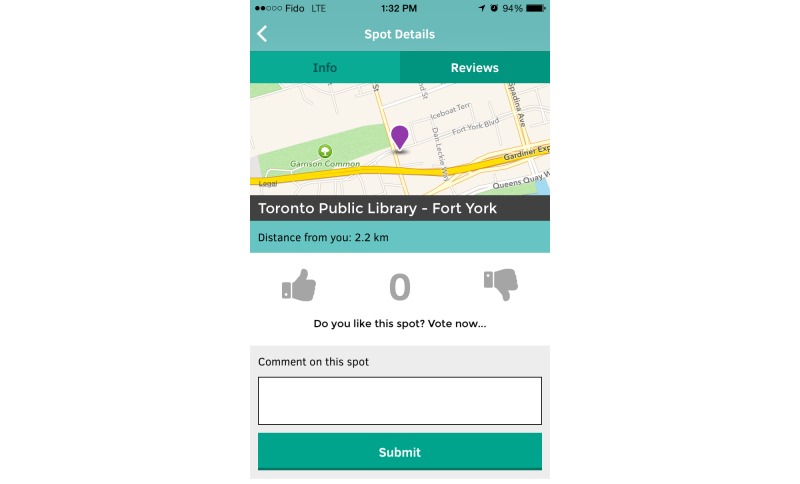
Users can post reviews about spots. These reviews will be available to others who may be interested in accessing that resource.

**Figure 6:**
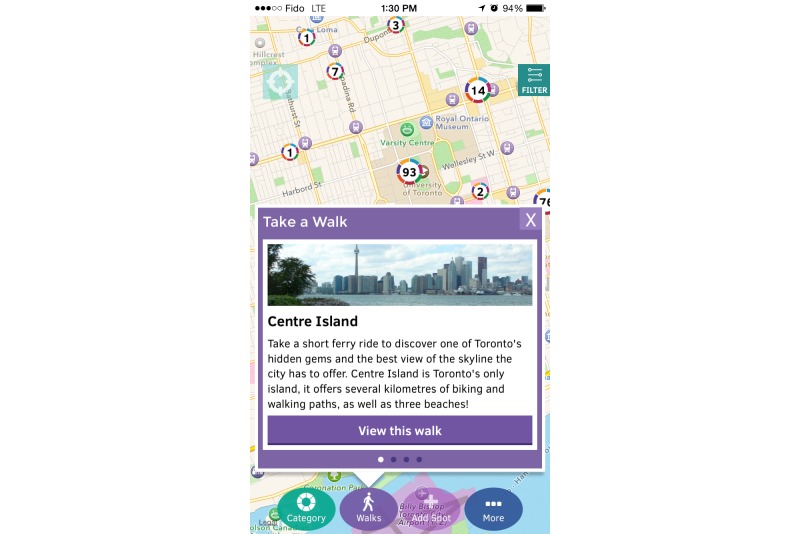
There are predesigned routes designed for users to take walks around their neighbourhood to increase their awareness of the resources available.

Additional functionality includes crowd-sourcing, an increasingly important data-gathering technique that allows users of a content-rich resource to submit revised and/or new information that, after vetting, is fed back into the resource [54]. Thus, Thought Spot users can add additional *spots*, allowing them to grow the platform and shape it according to their needs and interests (Figure 4). This built-in crowd-sourcing functionality also includes the ability for users to give a simple *thumbs up* or *thumbs down* rating to individual services, and to make comments about particular services (Figure 5). Finally, building on an idea that came from the winning entry from the *Hackathought* event, users can find *wellness walks* built into the map (Figure 6). These routes are intended to reinforce the overall wellness focus of Thought Spot by encouraging students to take healthy breaks from their studies and other activities, and to explore the city in which they are studying, and become more connected with its communities. A functional Web version of Thought Spot [55] and a mobile app (for iOS and Android) are readily available online via free download. These platforms have gone through a series of usability testing sessions with student participants, using the state-of-the-art facilities and expertise at the University Health Network (UHN) Centre for Global eHealth Innovation. The current state of the platform has been assessed at Technology Readiness Level 6 [56].

### Optimization Process and Research Methodology

The current study to optimize the Thought Spot platform will be conducted in two distinct phases, which will aim to (1) optimize the Thought Spot Web and mHealth platform through youth engagement, and (2) evaluate the impact of Thought Spot on self-efficacy for mental health help-seeking and health literacy among university and college students through a randomized controlled trial (RCT).

Participants will be recruited across 3 participating postsecondary institutions in the GTA: University of Toronto, Ryerson University, and George Brown College, which have respective enrolments of 84,556, 38,000, and 31,187 students [57-59]. Recruitment strategies will include invitation letters on public university bulletin boards, health and technology academic department listservs, student groups, Residence Life staff, and social media (Twitter, Facebook, and Instagram). All participants will be required to sign a consent form prior to being recruited into either phase of the study. Once participants have provided informed consent, they will fill out a nonidentifiable demographics form. This demographic information will be used for comparison purposes, reporting findings, and preparing formal evaluation reports.

To take part in Phase 1 of the project, participants must be transition-aged youth (16-29 years) [60] of any gender who are currently enrolled in part-time or full-time studies at any Canadian postsecondary institution. To take part in Phase 2, participants must be transition-aged youth (16-29 years) [60] of any gender who are currently enrolled in part-time or full-time studies of one of the 3 participating postsecondary institutions. All participants must have functional competency in English. Phase 2 participants must also currently be seeking mental health support or have a self-identified need for mental health support, and must have access to digital devices compatible with the Thought Spot digital platform. Participants who self-identify as having been hospitalized within the last 3 months for a mental health or addictions concern will be excluded from both phases of the study.

### Phase 1: Optimization of mHealth Intervention

The Thought Spot platform and its implementation will be optimized using a model of engagement in which transition-aged youth attending colleges and universities colead all aspects of the project [60]. Students will have ownership of process, product, and deliverables. The engagement goal is to create a context in which students draw on their strengths and lived experiences, and in the process increase their health literacy, reduce mental health stigma, and become advocates for good mental health. The research question for Phase 1 is: *How can the Thought Spot intervention be optimized to enhance its current functionality, and increase the potential for its sustainability and adoption among postsecondary students, through youth engagement?*

The goals of Phase 1 are to (1) optimize the Thought Spot platform to more effectively meet the needs of end-users; (2) explore students’ motivations and identify personal, behavioral, and environmental influences that will affect their uptake of the Thought Spot platform; and (3) assess Thought Spot’s feasibility, usability, and acceptance by the target population.

#### Participatory Action Research and Participatory Design Research

Through the use of participatory action research (PAR) principles, students across the GTA will be recruited and engaged to drive the optimization of the Thought Spot platform. PAR focuses on improving health and reducing health inequities by involving the target population via an iterative cycle of reflection, data collection, and action [61,62]. The research is initiated and shaped with people affected by a specific issue, in partnership with academic researchers [61-65]. This PAR strategy will encompass different levels of involvement, from passive participation to self-mobilization. Participation incentives will vary depending upon the individual’s role and involvement.

Participatory design research (PDR) will also be employed. PDR is typically conducted by technology companies to allow for the direct involvement of the target audience in codesigning the technologies that they use. This design model is critical to gaining credibility among transition-aged youth [66]. This study will utilize various strategies, identified in the framework for *Participatory Design of Evidence-based Online Youth Mental Health Promotion, Prevention, Early Intervention, and Treatment* [67], to engage with students to codesign and inform various aspects of the project. These strategies will include codesign workshops (prototyping), usability testing, and focus groups, as described in the framework [67].

#### Research Process

Using PAR and PDR techniques, students will cocreate relevant engagement activities with the research team to obtain information from the target audiences, in order to optimize the Thought Spot platform and inform the second phase of this project. Students will be provided with relevant training on research methodology so that they can fully participate throughout the research process. Nonstudent facilitators and researchers will also be involved in this youth engagement process, and will be present during the engagement activities.

Students will be involved in the following activities in Phase 1 of the project:

1. Project oversight and input as a member of the Thought Spot Student Group (TSSG) and/or various subworking groups.

2. Engagement activities to assess the feasibility, usability, and acceptability of the intervention, and to inform the optimization of Thought Spot and potential outcomes.

3. Crowd-sourcing workshops (online and in person) to update and verify the resources on the Thought Spot map.

Students will also cocreate and provide feedback on various aspects of Phase 2 of the project, including outcome measures, recruitment and engagement strategies, study administration, and knowledge translation and dissemination strategies.

Engagement activities will be developed and facilitated with consenting members of the TSSG to explore our research questions and gain input from additional students. These engagement activities may include focus groups, interviews, and design workshops. A sample question guide has been developed by the research team for the engagement activities, but it will be modified (with the students) for each engagement activity prior to implementation (Table 1). We will also assess Thought Spot’s feasibility, usability, and acceptance by the target population, using the USE (usefulness, satisfaction, and ease-of-use) Questionnaire [68].

We may also use the various scales (eg, help-seeking scale, mental health self-efficacy scale) intended to be implemented in the RCT phase to inform questions asked during engagement activities, and to gather reflections from students about outcomes.

**Table 1:** Sample question guide developed by the research team for engagement activities.

Parameter	Question
Help-Seeking	How is accessing mental health services, or asking for mental health or substance use support, perceived by your friends?
	Have you ever searched for mental health, substance use, or wellness services for yourself or for someone else? What was your experience looking for this information?
Motivation/Uptake	What impact do you think Thought Spot could have on how students look for help?
	What are some barriers or challenges that might prevent you from using Thought Spot?
Optimization and Usability	What would you add or change to the app to make it more likely for you, or other students, to use Thought Spot?
	What changes, or enhancements, do you think will impact how students look for help?
Recruitment	We are planning to host a series of codesign workshops to help us improve Thought Spot. Codesign workshops allow students to work with researchers as partners in the development and refinement of products. What are the best methods to advertise to, or recruit, students for the codesign workshops? Why?

Note-takers will be present during all activities to document the discussions and outcomes. Flip charts and any other documentation will be collected and collated. Audio recorders will be used to record small group discussions. All audio recordings will be transcribed and analyzed.

During Phase 1 of the study, we anticipate that several enhancements will be identified to increase Thought Spot’s functionality, interactivity, and crowd-sourcing ability, in preparation for the implementation phase (Phase 2). We will explore the acceptability and feasibility of incorporating coaching or peer mentorship within the platform. The prototype will also be integrated with evidence-based consumer health literature sourced from Portico, CAMH’s online portal, and elsewhere. Working with the *Journal of Medical Internet Research* (JMIR), we will also explore the potential of linking Thought Spot with other evaluated online mental health interventions and mobile apps. Our technology partner, Quality of Care (QoC) Health, will develop the newly optimized version of Thought Spot and provide support for quality assurance and secure infrastructure. The prototype will be enhanced to include more robust user analytics and feedback mechanisms. Several enhancements will be made using QoC Health’s technology Engagement Platform (Technology Readiness Level 9) [56].

#### Ethical Considerations

Student participants will be given training and support through the project to ensure adequate research skills, mental health and addictions knowledge, and support regarding self-care, coping and stigma [69]. We will actively encourage students with lived experiences of mental health concerns and/or substance use to participate through our recruitment efforts. However, participants will not be asked to publicly disclose or discuss their mental health status at any point throughout the engagement activities. We will be engaging vulnerable individuals in this process, and protocols will be put in place to monitor and respond to participants’ needs at various stages in the study. Access to appropriate mental health crisis support will be identified for each university or college recruitment site, and information regarding services will be provided to all participants. Key members of the research team will be able to provide emergent clinical input or direction, should the need arise. These members of the research team will also provide direction and training to ensure appropriate, meaningful, and effective engagement with youth, especially vulnerable youth.

#### Analysis Plan

Data gathered during the various engagement activities will be thematically analyzed by qualified members of the research team and interested students. These data sets will be used to answer the research questions and inform Phase 2 of the project (ie, the RCT). We will use a thematic analysis process [70] to review the collected data, generate codes, develop themes, and present the findings in a final report. Collated notes and transcripts will be provided to students, to verify their accuracy and elicit feedback on the analysis.

A descriptive statistical analysis will be conducted on all sociodemographic responses. Acceptance and feasibility will be analyzed using the USE Questionnaire [68] *.* Means and standard deviations for each category of the questionnaire will be analyzed. The extent of participants reporting high, medium, or low levels of satisfaction on the USE Questionnaire will also be calculated.

Using the study results, we will identify specific enhancements to the Thought Spot platform. We anticipate enhancements will include (1) user analytics and feedback mechanisms, (2) peer mentorship and/or coaching functionality, (3) crowd-sourcing and data hygiene, and (4) integration of evidence-based consumer health and research information. Modifications will be made to the platform by QoC Health, with oversight provided by the TSSG and the research team. The modified platform will undergo usability testing at the UHN Centre for Global eHealth Innovation.

We will also use gender-based analyses of the data in both phases. Mental illness and substance use disorders develop and present in different ways, and at different rates, for young females and young males. For example, young females aged 15-24 years have higher rates of depression than their male counterparts (9% vs 5.3%); however, young males are more likely to suffer from substance use disorders than young females (4.7% vs 1.7%) [71]. Data regarding participants’ gender identities and orientations will be voluntarily and confidentially collected at intake. Results will be analyzed to assess similarities and differences between and among gender groups. This comparison will help us to identify unique help-seeking needs and patterns to better tailor the intervention, and ensure it is inclusive of all gender identities and sexual orientations.

### Phase 2: Randomized Controlled Trial

An RCT, with 2 arms, will be conducted in Phase 2 to assess the impact of Thought Spot on self-efficacy for mental health help-seeking and health literacy among our target population. Students in the intervention arm will have access to the Thought Spot platform (online and mobile versions), while the control arm will receive usual care (access to campus health services, Web- and print-based information materials). The study will last 6 months. Participants in the intervention arm will receive an in-person tutorial on how to use Thought Spot, as well as access to an online *tour the app* video. Data will be collected from participants through online questionnaires at baseline, 3 months, and 6 months. The full procedure regarding the design of the RCT and administration of the intervention will be codeveloped with students in Phase 1.

The main outcome measure will be changes in help-seeking intentions, measured by the General Help-Seeking Questionnaire (GHSQ), which will be administered at baseline, 3, and 6 months. This scale is composed of 10 items measured in a 7-point Likert scale that ranges from *Extremely Unlikely* to *Extremely Likely*. The items are sources of help (eg, parent, friend, family doctor) and the participants are asked their likelihood of seeking help from each source. The 10 items are usually split into *informal help sources*, *formal help sources*, and *I would not seek help from anyone*.

We will also use the Actual Help-Seeking Questionnaire (AHSQ) and Attitudes Toward Seeking Professional Psychological Help Scale-Short Form (ATSPPH-SF) to measure secondary outcomes: help-seeking behaviors and help-seeking attitudes. These scales are most often used in help-seeking studies [72], and have been used in similar studies and with similar populations, often together [13,22,73,74].

Adequate internal consistency has been reported for the GHSQ in studies of high school students aged 12-19 years (Cronbach alpha range of .70–.90) [75,76] and university students (Cronbach alpha=.67) [77], as well as adequate validity and very good test-retest reliability (*r*=.86) [75]. The correlation between intentions to seek mental health care for personal-emotional problems and actually seeking care has been found to be positive and significant for the GHSQ (*r* s[218])=.17, *P*<.05). The ATSPPH-SF has demonstrated good internal consistency (Cronbach alpha range of 0.82–0.84), 1-month test-retest reliability of 0.80, and a correlation of 0.87 with the longer ATSPPH scale, among samples of college students [78-80].

In addition to these scales, we also plan to explore self-reported changes in self-stigma using the Self-Stigma of Seeking Help Scale [81] and self-efficacy using the Youth Efficacy/Empowerment Scale - Mental Health [82]. We will also gather data on demographics and general mental health status (Global Appraisal of Individual Needs - Short Screener, Canadian/CAMH version) [83] to examine trends, comparisons, and correlations between the study groups.

#### Power Analysis

The outcome considered for our power calculation is the average of the GHSQ scale for the formal sources, although similar effect and sample sizes are expected for the informal sources.

To determine the sample size required to test the primary hypothesis that the Thought Spot intervention will cause a greater change in help-seeking intentions than usual care, a series of Monte Carlo simulations were carried out (with 10,000 replications under each test scenario) using SAS System 9.4 for Windows [84]. These simulations assume that the primary hypothesis will be tested using mixed-effect models to account for the longitudinal design, and linear contrasts between time and arm to compare the changes from baseline in both arms. We assume that the test will be 2 tailed and with a critical Cronbach alpha level of 0.05. In order to simulate the data, the means, standard deviations, and within-subject associations are assumed to follow published data that have used similar study designs [73,74]. Based on previous research using the GHSQ [47], a small effect size, equivalent to a Cohen’s d of 0.25, was considered (ie, an average change in the GHSQ of 0.41, which is equivalent to a change of 15%), within-subject correlation of 0.6, attrition rate of 40%, and power of 80%. Based on these simulations and specifications, a sample of 236 subjects per arm at baseline is required, which amounts to 472 subjects in total. If 40% attrition is applied to this initial sample, we will be left with 142 subjects per arm (283 in total) after 9 months, at the conclusion of the study.

#### Statistical Analyses

All analyses will be carried out using SAS System 9.4 for Windows. Statistical tests will be 2 sided, with confidence levels of 0.05. Prior to testing, a series of univariate analyses will be carried out to ensure that model assumptions are met. To address the primary study hypothesis, a mixed-effect model will be used to account for the longitudinal nature of the data, and for attrition. Missing values will be treated with maximum likelihood estimation in SAS PROC MIXED, which uses all available information in the data. Intention-to-treat analysis will be used; all patients will be analyzed as they were originally allocated after randomization. As a sensitivity analysis, the final model will be fitted only with subjects for whom there is complete data. Formal help-seeking score will be the dependent variable, with study groups (intervention and control) and time points as predictors, and relevant sociodemographics collected at baseline as covariates to control for possible confounding. The interaction between study group and time will be included in the model, and linear contrasts will be used to compare the groups, specifically regarding the change from baseline to the final time point. Similar models will be used to address the exploratory hypotheses, which examine different scales and trends, on the effect of the intervention over time. Bonferroni adjustment will be used to control the Type I error rate if multiple comparisons are desired. Generalized estimating equations will be used for the AHSQ, since this scale is binary.

### Other Analyses

A sex- and gender-based analysis will be completed when analyzing data for Phases 1 and 2. An economic evaluation of the Thought Spot intervention compared to usual care will also be explored to determine the potential cost-effectiveness and financial implications of sustainable and widespread use of Thought Spot throughout Canadian postsecondary campuses. The evaluation will be conducted from the perspective of future potential Thought Spot funders (eg, other Canadian postsecondary institutions). The primary outcome to be assessed will be the change in helping-seeking intentions among the target population.

### Approach to Bias Control

We are using computer-generated random allocation of subjects to the intervention and control arms, by means of the Research Randomizer website [85]. This will be a partially blind study, in that the process of inviting students and collecting data will not be done by the researchers. Missing values will be minimized by sending reminders to participants at least 3 times, and the offer of an honorarium at the end of the fourth data collection point. It is possible that students in the control group may use Thought Spot, so we will ask control subjects at the end of the final survey whether they accessed Though Spot during the study. This approach will allow us to carry out a sensitivity analysis to exclude control subjects who accessed Thought Spot. To mitigate this possibility, we will investigate password protecting the platform or tracking email addresses through a sign-in process during the trial period. We cannot guarantee that control subjects will not interact with intervention subjects, causing some contamination, but given the personal nature of mental health conditions, we believe this risk will not be highly prevalent.

Certain members of the research team were involved in the development of the original Thought Spot platform. To avoid bias or perception of bias, the scientific lead for the project, who was not involved in the development of the original Thought Spot platform, will oversee the RCT.

### Hypotheses: Phase 2

We hypothesize that transition-aged youth who receive the intervention will show a greater improvement in intentions for, and self-efficacy in, help-seeking for mental health concerns, compared to those who are allocated to the control group (usual care; resource pamphlet). We also hypothesize that participants in the intervention arm will show greater improvements in health literacy, increased self-efficacy in managing their mental health concerns, and a reduction in mental health stigma, compared with the control arm.

## Results

Phase 1 of this study is currently underway and will continue until August 2017. It is anticipated that this phase of the study will result in the optimization of Thought Spot and provide important information for refining Phase 2 of the study. The results of Phase 1 will be based on principles of cocreation and coproduction, and will be guided by PAR and PDR.

Phase 2 will be carried out between September 2017 and September 2019. Phase 2 will allow us to test our primary hypothesis that transition-aged youth who receive the intervention will show a greater improvement in intentions for, and self-efficacy in, help-seeking for mental health concerns. Results will be available following the conclusion of each phase.

## Discussion

Research indicates that eHealth and mHealth interventions for youth mental health should provide information in 3 primary areas: positive mental health or mental wellness; mental illness (eg, myths, symptoms); and help-seeking strategies and methods for accessing mental health services [24]. Transition-aged youth have reported that they want help in a number of areas: determining if there is a mental health problem, and finding support or help; being empowered by the provision of health information, without an intermediary [35]; and the ability to connect with peers [24].

Transition-aged youth generally trust online sources for health advice [86], and university students are likely to first seek help online [28]. At the time of one study, almost one-third (31%) of teenagers (ages 12-17) and 72% of young adults (ages 18-29) were found to seek health information online, including mental health information on issues such as drug use and depression [87]. The ongoing development of our Thought Spot platform, in a way that is cocreated and crowd-sourced, will inform and contextualize the design and methods for the target population, and may influence the efficacy of the intervention.

Transition-aged youth living with a mental illness are more likely than those not living with a mental illness to report engaging in various social networking activities that promote connectivity, anonymity, and making online friends, and activities that enable independent living skills and overcoming social isolation [87,88]. A recent scoping review of youth mental mHealth interventions found that the “flexibility, interactivity, and spontaneous nature” of mHealth interventions encouraged “persistent and continual access to care outside of clinical settings” for transition-aged youth [89]. Emerging evidence suggests that a *coach* (peer or professional) can improve outcomes using computer-based and mHealth interventions without the need for an intermediary such as parents, a physician, or other service providers [35]. To this end, we expect Thought Spot to be a powerful tool to bridge the health literacy gap, and facilitate appropriate help-seeking for this population.

The open-source crowd-sourcing software used in this project allows the Thought Spot map to be scalable to any jurisdiction, and adapted as needed. While the intervention will initially be implemented and evaluated within the GTA, the reach of Thought Spot can be expanded to other interested jurisdictions. Using an open-source crowd-sourced approach allows significant potential to scale this project to a national and international level. Integrating the existing Thought Spot technology into QoC Health will allow for a rapid scale-up with its robust team, user-centered approach to design, and strategic partnerships. The ability to crowd-source within Thought Spot will be open to students and to any service provider that has been mapped (or wants to be mapped), and is key to the currency and sustainability of the resource. Crowd-sourced data within Thought Spot will be monitored continuously through the platform, and will be verified and self-corrected by end-users themselves.

Thought Spot has the potential to be implemented or scaled in provincial, national, and international contexts. This platform provides a significant opportunity to address the information- and help-seeking needs of transition-aged youth by integrating access to consumer health information, pulling from both CAMH’s existing portfolio of public information (including a significant stock of information already developed for youth populations) and an array of other, high-quality, publicly accessible information.

### Conclusions

This protocol outlines the important next steps in understanding the role of the Thought Spot platform on the behavior of postsecondary, transition-aged students when seeking information and services related to mental health and addiction issues. Phase 1 of the study will allow for the continuation of cocreating and coproducing a solution that will be further optimized to meet the needs of its target population. The first phase will have the added benefits of potentially raising awareness of mental health issues, and providing an opportunity for students to become involved in ensuring that their peers have timely access to appropriate services. Phase 2 of the study will explore the efficacy of the crowd-sourced platform in improving students’ sense of self-efficacy and their ability to seek appropriate information and services in a timely manner. The results of the RCT study will further contribute to our understanding of how best to support postsecondary, transition-aged students who are seeking information and support related to mental health and addiction concerns.

## Abbreviations

AHSQ: Actual Help-Seeking Questionnaire
ATSPPH-SF: Attitudes Toward Seeking Professional Psychological Help Scale - Short Form
CAMH: Centre for Addiction and Mental Health
eHealth: electronic health
GHSQ: General Help-Seeking Questionnaire
GTA: Greater Toronto Area
JMIR: Journal of Medical Internet Research
mHealth: mobile health
OCAD: Ontario College of Art and Design
PAR: participatory action research
PDR: participatory design research
QoC: Quality of Care
RCT: randomized controlled trial
TSSG: Thought Spot Student Group
UHN: University Health Network
USE: usefulness, satisfaction, and ease-of-use

## Appendix

Multimedia Appendix 1 CIHR reviewers comments, which have been taken into account within the protocol.
