# Supplementary material for: Enhancing Self-Efficacy for Help-Seeking Among Transition-Aged Youth in Postsecondary Settings With Mental Health and/or Substance Use Concerns, Using Crowd-Sourced Online and Mobile Technologies: The Thought Spot Protocol
Source: JMIR Res Protoc. 2016 Nov 4;5(4):e201. doi: 10.2196/resprot.6446 (PMC5116103; doi:10.2196/resprot.6446)
Supplement: Multimedia Appendix 1 [file resprot_v5i4e201_app1.pdf]

October/Octobre 30, 2015

DC0190GP

Canadian Institutes of Health Research / Instituts de recherche en santé du Canada

Notice of Decision / Avis de décision

Committee Code/Code du comité: EH1

Applicants/Candidats: Dr. David E. Wiljer

With/Avec:				
	Ms. A. Abi-Jaoude	Ms. G. Chaim	Dr. K. Cleverley	Prof. G. Eysenbach
	Dr. J. Henderson	Dr. J. Hoch	Dr. W. Isaranuwatchai	Dr. H. Jiang
	Dr. M. Law	Dr. A. Levinson	Ms. J. Robb	Dr. A. Voineskos

Institution paid/ Établissement payé: Centre for Addiction and Mental Health (Toronto)

Title/Titre: ThoughtSpot: Enhancing self-efficacy for help-seeking among transition-aged youth in postsecondary settings with mental health and/or substance use concerns using crowd-sourced online and mobile technologies.

Primary Inst./ Inst. principal: Human Development, Child and Youth Health

Other Related Inst./ Autres inst. connexes: Health Services and Policy Research; Population and Public Health

Competition Outcome/Résultats du concours: Oper Grant:eHealth Innov Partnership Program(eHIPP)-Youth&Adolescent Mental Hlth
June/Juin 03, 2015

Number in competition/Nbre de demandes dans le concours: 16

Number approved/Nbre de demandes approuvées: 8

Decision on your application/ Décision sur votre demande: Approved

Average annual amount/ Montant annuel moyen: $187,495

Equipment amount/ Montant pour les appareils: $0

Term/Durée: **4 yrs/ans 0 months/mois**

Peer Review Committee Recommendation, for your information and use/
Recommandation du comité d'examen par les pairs, pour fins d'information et d'utilisation:

Committee/Comité: eHealth Innovation Partnership Program (eHIPP): Youth and Adolescents Mental Health

Application rank within the competition/ Rang de la demande dans ce concours: 3

Percent Rank Within the Competition/ Rang en pourcentage au sein du concours: 18.75%

Rating/ Cote: 4.11

Recommended average annual amount/ Montant annuel moyen recommandé: $187,495

Recommended equipment amount/ Montant recommandé pour les appareils: $0

This document is for information only. Official payment is stated on the CIHR Authorization for Funding.
Document à titre d'information seulement. Le paiement officiel est indiqué sur l'autorisation de financement des IRSC.

**Review Type/Type d'évaluation:**	SO Notes /Notes de l'agent scientifique
**Name of Applicant/Nom du chercheur:**	WILJER, David E.
**Application No./Numéro de demande:**	348307
**Agency/Agence:**	CIHR/IRSC
**Competition/Concours:**	2015-06-03 Operating Grant: eHealth Innovations Initiative: eHealth Innovation Partnership Program (eHIPP)/Subvention de fonctionnement: Initiative Innovations en cybersanté : Programme de partenariats pour l'innovation en cybersanté (PPIC)
**Committee/Comité:**	eHealth Innovation Partnership Program (eHIPP): Youth and Adolescents Mental Health/Programme de partenariats pour l'innovation en cybersanté-Des jeunes et la santé mentale
**Title/Titre:**	ThoughtSpot: Enhancing self-efficacy for help-seeking among transition-aged youth in postsecondary settings with mental health and/or substance use concerns using crowd-sourced online and mobile technologies.

Assessment/Évaluation:

The application proposes using crowd-sourced online and mobile technologies to enhance help-seeking among transition-aged youth in post-secondary settings. The intervention proposed was highly innovative in its use of crowdsourcing of information from young people. Another strength was the involvement of youth in decision-making and development of the project. The team is strong and feasibility of implementation is high. There was some concern about the commercialization potential of the innovation. The evaluation of the intervention was a weakness, in particular using service use as an outcome variable rather than the mental health and well-being of the users.

**Review Type/Type d'évaluation:**	Committee Member 1/Membre de comité 1
**Name of Applicant/Nom du chercheur:**	WILJER, David E.
**Application No./Numéro de demande:**	348307
**Agency/Agence:**	CIHR/IRSC
**Competition/Concours:**	2015-06-03 Operating Grant: eHealth Innovations Initiative: eHealth Innovation Partnership Program (eHIPP)/Subvention de fonctionnement: Initiative Innovations en cybersanté : Programme de partenariats pour l’innovation en cybersanté (PPIC)
**Committee/Comité:**	eHealth Innovation Partnership Program (eHIPP): Youth and Adolescents Mental Health/Programme de partenariats pour l'innovation en cybersanté-Des jeunes et la santé mentale
**Title/Titre:**	ThoughtSpot: Enhancing self-efficacy for help-seeking among transition-aged youth in postsecondary settings with mental health and/or substance use concerns using crowd-sourced online and mobile technologies.

Assessment/Évaluation:

The crowd sourcing element of this proposal is innovative
The potential to influence help-care seeking is worthy of systematic evaluation

**Review Type/Type d'évaluation:**	Committee Member 2/Membre de comité 2
**Name of Applicant/Nom du chercheur:**	WILJER, David E.
**Application No./Numéro de demande:**	348307
**Agency/Agence:**	CIHR/IRSC
**Competition/Concours:**	2015-06-03 Operating Grant: eHealth Innovations Initiative: eHealth Innovation Partnership Program (eHIPP)/Subvention de fonctionnement: Initiative Innovations en cybersanté : Programme de partenariats pour l'innovation en cybersanté (PPIC)
**Committee/Comité:**	eHealth Innovation Partnership Program (eHIPP): Youth and Adolescents Mental Health/Programme de partenariats pour l'innovation en cybersanté-Des jeunes et la santé mentale
**Title/Titre:**	ThoughtSpot: Enhancing self-efficacy for help-seeking among transition-aged youth in postsecondary settings with mental health and/or substance use concerns using crowd-sourced online and mobile technologies.

Assessment/Évaluation:

Excellent application. Amazing use of the technology - innovative and creative.

Review Type/Type d'évaluation: Committee Member 3/Membre de comité 3
Name of Applicant/Nom du chercheur: WILJER, David E.
Application No./Numéro de demande: 348307
Agency/Agence: CIHR/IRSC
Competition/Concours: 2015-06-03 Operating Grant: eHealth Innovations Initiative: eHealth Innovation Partnership Program (eHIPP)/Subvention de fonctionnement: Initiative Innovations en cybersanté : Programme de partenariats pour l'innovation en cybersanté (PPIC)
Committee/Comité: eHealth Innovation Partnership Program (eHIPP): Youth and Adolescents Mental Health/Programme de partenariats pour l'innovation en cybersanté-Des jeunes et la santé mentale
Title/Titre: ThoughtSpot: Enhancing self-efficacy for help-seeking among transition-aged youth in postsecondary settings with mental health and/or substance use concerns using crowd-sourced online and mobile technologies.

Assessment/Évaluation:

The ThoughtSpot project seems to have a good collaborative network between OCAD, Ryerson, University of Toronto, ConnexOntario, and Kids Help Phone. The name itself is great as it is not stigmatizing and is also a very accurate description of what the project is. Something I really appreciate is the inclusion of so many youth in the development of the initiative, including the representatives on the team. I also think there is a lot of potential based on the support of numerous university officials, given the target audience.

I am not, however, entirely certain that ThoughtSpot will be very transformative. Both from my experience and from what I've gathered from peers who are also patients, the demand doesn't seem to be so much for information. After all, information seems to be readily at our fingertips, and postsecondary students develop research skills as a result of their academics—these skills can easily be applied to investigating mental health services. If anything, the demand is for more access to those services, and I do not know if ThoughtSpot will really meet that demand.

Review Type/Type d'évaluation: Committee Member 4/Membre de comité 4
Name of Applicant/Nom du chercheur: WILJER, David E.
Application No./Numéro de demande: 348307
Agency/Agence: CIHR/IRSC
Competition/Concours: 2015-06-03 Operating Grant: eHealth Innovations Initiative: eHealth Innovation Partnership Program (eHIPP)/Subvention de fonctionnement: Initiative Innovations en cybersanté : Programme de partenariats pour l'innovation en cybersanté (PPIC)
Committee/Comité: eHealth Innovation Partnership Program (eHIPP): Youth and Adolescents Mental Health/Programme de partenariats pour l'innovation en cybersanté-Des jeunes et la santé mentale
Title/Titre: ThoughtSpot: Enhancing self-efficacy for help-seeking among transition-aged youth in postsecondary settings with mental health and/or substance use concerns using crowd-sourced online and mobile technologies.

Assessment/Évaluation:

Synopsis:
The project will test an mHealth intervention prototype 'Thoughtspot' among transition-aged youth to determine whether there is an improvement in help seeking behaviours about their mental health concerns. Interactive Thoughtspot which helps to connect the user to mental health service providers has been developed by university and colege students is claimed to be user friendly. Thoughtspot researchers and developers envisage that the mHealth intervantion will help to encourage 70% of transition-aged youth (before they become 24 years old) to seek health and well being services and mitigate the lack of availability mental health care services in terms of mental health or wellness literacy, improve help seeking attitude and reduce the stigma associated with mental illness .

Planned Intervention:
- Technology Readiness Level (TRL): proof of concept provided. A mhealth intervention prototype 'ThoughtSpot' has been by university students.
- The functionality/system components of the open-source platform, alongside with the enhancements/additional functions this proposed plans to implement need to be provided. Lack of this information make it hard to evulate the innovation of the proposal.
- While the need for holistic approach towards technology design is outlined , its three components, namely, preventative care, proactive care and reactive care and technological design around these three pillars has not been clearly elucidated.
Technology role is seen more as an information provider rather than a problem solver. Technological innovation is low to medium.
eHealth Innovation Evaluation:
-Although usability testing is budgeted, there is lack of detail on the measures that will be used to test usability and their link to other outcome measures. No measures developed around capture and analysis of non-verbal information for purpose of usability and self-efficacy

Team:
- The team is well structured and has prior experience in working together. Too much information about the technology partern, but information about the scientific team is limited.-

**Review Type/Type d'évaluation:**	Committee Member 4/Membre de comité 4
**Name of Applicant/Nom du chercheur:**	WILJER, David E.
**Application No./Numéro de demande:**	348307
**Agency/Agence:**	CIHR/IRSC
**Competition/Concours:**	2015-06-03 Operating Grant: eHealth Innovations Initiative: eHealth Innovation Partnership Program (eHIPP)/Subvention de fonctionnement: Initiative Innovations en cybersanté : Programme de partenariats pour l'innovation en cybersanté (PPIC)
**Committee/Comité:**	eHealth Innovation Partnership Program (eHIPP): Youth and Adolescents Mental Health/Programme de partenariats pour l'innovation en cybersanté-Des jeunes et la santé mentale
**Title/Titre:**	ThoughtSpot: Enhancing self-efficacy for help-seeking among transition-aged youth in postsecondary settings with mental health and/or substance use concerns using crowd-sourced online and mobile technologies.

Assessment/Évaluation:

Budget:
There is important in-kind contribution in through the development of Thoughtspot prototype. Budgeted items are in the right areas with adequate justification. However, in reviewer's opinion given they have already tested the prototype which is functional since September 2014 why funding is required for 4 years and not say 2 or 3 years to complete design and deployment of security related programs, customization and usability testing. Maybe the team should complete the project in 3 years.
